# A qualitative study to identify the determinants and strategies for the prevention of dengue fever in Iran

**DOI:** 10.1038/s41598-025-11423-x

**Published:** 2025-08-14

**Authors:** Razie Toghroli, Abdoljabbar Zakeri, Mehdi Hassani Azad, Minoosadat Mousavi Nik, Roghayeh Ezati rad, Zohre Eftekhari, Mehdi Sharafi, Manoj Sharma

**Affiliations:** 1 Present Address: Social Determinants in Health Promotion Research Center, Hormozgan Health Institute, Hormozgan University of Medical Sciences, Bandar Abbas, Iran; 2 Department of Community Medicine, School of Medicine, Hormozgan University of Medical Sciences, Bandar Abbas, Iran; 3 School of Medicine, Infectious and Tropical Diseases Research Center, Hormozgan Health Institute, Bandar Abbas, Iran; 4 Present Address: Surgical Oncology Research Center, Mashhad University of Medical Sciences, Mashhad, Iran; 5 Fertility and Infertility Research Center, Hormozgan University of Medical Sciences, Bandar Abbas, Iran; 6 Biotechnology Department, Pasteur Institute of Iran, Tehran, Iran; 7 Department of Social and Behavioral Health, School of Public Health, University of Nevada Las Vegas (UNLV), Las Vegas, NV, 89119, United States; 8 Cellular and Molecular Research Center, Gerash University of Medical Sciences, Gerash, Iran

**Keywords:** Dengue fever, *Aedes* mosquito, Prevention and control, Social participation, One health, Qualitative research, Public health, Health care, Health services

## Abstract

In June 2024, Iran reported a dengue fever outbreak beginning in Hormozgan province and spreading to several other provinces, raising significant public health concerns. To mitigate the disease’s impact, multiple meetings were held to explore control strategies, emphasizing community participation through focus groups. This study aimed to identify the determinants, priorities, and control strategies for combating dengue fever and breeding sites of Aedes mosquitoes in Hormozgan province, Iran. This qualitative study employed purposive sampling with maximum variation to conduct 13 focus group discussions (FGDs) with 163 participants (8–13 per group) in Hormozgan Province during June-July 2024. Participants included health department officials, municipal managers, port authorities, and community leaders. Data were analyzed using thematic analysis following Braun and Clarke’s six-phase approach, with trustworthiness ensured through member checking and peer debriefing. These FGDs included health department officials, governors, municipal managers, medical university representatives, Shipping offices, and influential community leaders involved in dengue prevention education. Through these discussions, seven key determinants for dengue fever control in Iran were identified. The primary factors were: (a) Environmental (b) Therapeutic and health care (c) interdisciplinary cooperation (d) Administrative, legal, and regulatory determinants (e) Financial and budgetary (f) Educational determinants and (g) Social determinants. Effective community empowerment and health program decision-making require cooperation across various organizations, enhancement of high-risk environments, and fostering a sense of responsibility and participation among community members. Given the rise of re-emerging diseases globally, identifying their determinants is crucial for quick disease control in the region and for preventing global pandemics.

## Introduction

Dengue fever represents a significant public health challenge in over 100 tropical and subtropical nations, with the World Health Organization documenting an eightfold global increase in cases since 2000^1,2^. The disease currently threatens 40–50% of the world’s population, with projections suggesting this could rise to 60% by 2030 due to climate-driven expansion of Aedes mosquito habitats^3,4^. The clinical spectrum ranges from self-limiting febrile illness to severe dengue characterized by plasma leakage, hemorrhagic manifestations, and multi-organ failure^5,6^. Aedes mosquitoes, which transmit dengue and other arboviruses like Zika and chikungunya, have expanded their geographic range due to climate change, urbanization, and increased global travel^7,8^. The economic burden is substantial, with annual costs exceeding $8 billion in endemic countries^9,10^.

**Fig. 1:**
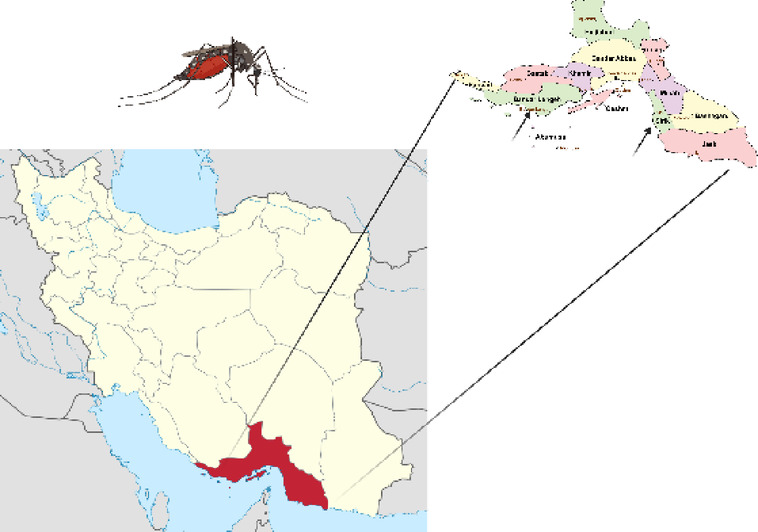
Geographical location of Bandar Lengeh (red dot) in Hormozgan Province, southern Iran. Identified as the initial epicenter of the dengue outbreak in June 2024, this port played a key role in virus introduction due to its maritime connections with endemic countries. Other major cities in the province are marked with black circles.

Hormozgan’s dengue emergence began when the first indigenous cases were identified in June 2024 in Bandar Lengeh—a strategic port city in Hormozgan province with extensive maritime connections to endemic countries like the UAE^11,12^(Fig. 1). Hormozgan’s tropical climate, busy international ports (including Shahid Rajaee, Iran’s largest container port), and high humidity create ideal conditions for Aedes albopictus, the secondary dengue vector now established in the region^13,14^. Despite the increasing prevalence, Iran faces significant challenges in surveillance, diagnosis, and vector control^15^. Similar to Saudi Arabia’s 2019 outbreak, Iran’s health system struggles with fragmented vector control programs and insufficient cross-border coordination^16,17^. The rapid spread to northern provinces like Gilan suggests climate change may be enabling vector adaptation beyond traditional tropical ranges^15,18^.

Prevention remains paramount as no specific antiviral treatment exists. Community-centered approaches greater compliance than punitive measures^19,20^. Successful models include Singapore’s “5-step Mozzie Wipeout” campaign and Thailand’s use of Gambusia fish for biological control^21,22^. However, Iran’s current reliance on reactive insecticide spraying is unsustainable. The country needs integrated strategies addressing environmental management (e.g., waste control), intersectoral collaboration, and community empowerment. Particular attention must be paid to port areas where international trade increases introduction risks^23–25^.

Existing studies in Iran focus primarily on entomological surveys rather than prevention frameworks^26,27^. Global research often neglects sociocultural contexts crucial for community engagement^28,29^. Our study addresses three critical gaps by^1^: analyzing outbreak determinants in hormozgan province as a sentinel site for climate-mediated disease emergence^2^, evaluating cost-effective adaptations of global prevention strategies for Iran’s unique context, and^3^ establishing multisectoral collaboration models applicable across climate transition zones. This study aimed to identify the determinants, priorities, and control strategies for combating dengue fever and breeding sites of Aedes mosquitoes in June and July of 2024 in Hormozgan province, Iran.

## Method

### Study design

This is a qualitative study with content analysis methodology. Since dengue fever control is an interdisciplinary issue, we used qualitative methods to explore why this epidemic occurred in Iran and to examine how it should be controlled by the interested organizations, to determine our current situation and control strategies to control and prevent this public health problem. We used a qualitative focus group discussion (FGD) study design. A focus group is a method that utilizes in-depth group interviews with participants chosen purposefully, although not always as a representative sample of a particular population. This group is ‘focused’ on a specific topic^30–32^. Participants are selected based on their ability to contribute to the discussion, falling within a certain age range, sharing similar socio-characteristics, and being at ease conversing with the interviewer and other participants. In the field of public health research, focus groups can be especially useful for understanding community-level barriers and facilitators of health behaviors, evaluating the acceptability and feasibility of proposed interventions, and gathering feedback to modify program design^30,33^.

### Participants

Following the confirmation of the first dengue fever case in Hormozgan province, linked to travel in the Persian Gulf region, the vice chancellor for the healthcare department promptly convened a series of urgent meetings. A total of 13 focus group discussions were conducted, each comprising between 8 and 13 participants, alongside a larger group meeting that included 20 individuals, totaling 163 participants from various stakeholder groups (Table 1). The inclusion criteria for this study were related to the issue of dengue fever control and involvement in the dengue fever control plan in Iran, and agreement to attend and participate in the study. Any unwillingness to continue participating in the study for any reason was an exclusion criterion from this study. The principle of data saturation was employed to conclude the FGDs, where the same themes started getting repeated.

Local health authorities and community leaders (deputy of health, deputy of education, deputy of treatment, municipality, governorates, environmental department, universities of medical sciences of the province), health care workers (such as physicians, nurses, community health workers), staff health deputy, community members from high-risk dengue fever regions (elders in rural areas and influential religious speakers in the region). To ensure diversity in age, gender, and socio-economic status of the participant’s, targeted sampling was done with maximum diversity. Participants were identified and invited to participate through administrative letters to relevant agencies and notification to local health authorities, social organizations, and door-to-door visits in high-risk neighborhoods. The participants gathered around each other at the Health Deputy of Hormozgan province, and semi-structured interviews started with general questions, followed by discussion and brainstorming.

**Table 1:** Demographic characteristics of the participants.

Variable		Absolute frequency(*N*)	Relative frequency (%)
Education	Diploma	18	11
	Bachelor’s degree	56	34
	Master of science	53	32.5
	General practitioner	21	13
	PhD	11	7
	Medical specialist	4	2.5
Employment	Health and care departments	69	42.5
	Governorate, municipality, and governorate offices	29	17.5
	The administration of shipping and ports	35	21.5
	The others	69	42.5
Gender	Male	106	65
	Female	57	35

### Data collection

Focus group discussion sessions were managed by a principal investigator with extensive experience in the relevant field and in conducting qualitative research. A trained moderator and facilitator, along with additional note-takers, assisted in documenting the sessions, which typically lasted between 85 and 130 min. With the participants’ consent, these sessions were audio recorded. Each session commenced with an introduction where the research objectives were outlined, and participants were encouraged to share their thoughts openly. They were assured that their responses would be recorded and published anonymously, emphasizing that all opinions were valuable and that there were no right or wrong answers. An interview guide was used to guide the sessions (Table 2).

**Table 2:** Interview guide.

Topics
1- Knowledge and understanding of dengue fever and strategies for its prevention
2- Understanding the current manners of society toward the prevention and control of dengue fever in different regions of the province
3- Understanding the challenges faced by stakeholders in dengue fever prevention and control implementation programs
4- Dengue fever situation in the country, the province, and the cities of Hormozgan province
5- Knowledge of the *Aedes* mosquito, including its identification and methods for protection against it.
6- Need assessment along with the prioritization of pertinent activities, taking into account the prevalence of disease, the specific type of region, and the urgency involved
7- Current community-based dengue control activities and their effectiveness
8- Obstacles and facilitators of community participation in dengue control
9- Recommendations for improving community participation in dengue prevention
10- Examining and concluding a memorandum of understanding between all relevant departments

### Data analysis

A thematic analysis was employed to identify and examine recurring patterns within the textual data. This thorough approach followed the six-phase analytic framework established by Braun and Clarke^34^.

(1) becoming familiar with the data, (2) generating initial codes, (3) identifying themes, (4) reviewing themes, (5) naming and defining themes, and (6) compiling the report.

### Rigor

Guba and Lincoln’s evaluation criteria were used to check the trustworthiness of the findings. To substantiate the validity of the findings, the researcher’s self-review technique was used in data collection and analysis, as well as a peer check during which the codes were provided to two participants to resolve misunderstandings. Also, long-term engagement with the data (3 month), attendance at and recording all sessions, both audio and written, confirms the credibility of the results. To evaluate and increase the validity of findings, the sampling continued until data saturation and a validation method was used by a number of participants. The data transfer was ensured by providing a comprehensive and complete description of the topic to the participants. In addition, they reviewed and confirmed the initial comments. Since all participants were from different occupational and social groups and were fully aware of the problems of individuals at the community level, the results obtained had good transferability. To confirm the reliability of the data, a detailed recording of the research process from sample selection to data analysis and recording of any changes in the research process was carried out by a number of researchers aware of the qualitative approach so that the data were free from single-researcher actions. Finally, to ensure the confirmability of the data, a combination of different methods for data collection and analysis (triangulation) was used, which included complete documentation of all data and providing the results to some participants and two external qualitative researchers for their confirmation for preventing any prejudice and bias[11]. In addition, complete documentation of the research process, from data collection to analysis and interpretation, is available at the Center for Research on the Prevention and Control of Infectious Agents at HUMS.

### Ethical considerations

This research was approved by the Ethics Committee of Hormozgan University of Medical Sciences (IR.HUMS.REC.1403.347). At the beginning of the group discussions, the main speaker tried to create a friendly atmosphere for the interview while introducing the researcher and also explaining the objectives of the research. Participants were also assured of the confidentiality of their personal information, the anonymity of the recorded conversations, and the reason for their selection. They consented to have their voices recorded. Participants were free to withdraw or leave the interview at any time. Findings will be disseminated to local health authorities, community organizations, and through peer-reviewed publications to inform the development of community-based dengue control strategies in Hormozgan Province. Written informed consent was obtained from individuals who participated in this study. The authors confirm that all methods were performed in accordance with the relevant guidelines and regulations.

## Results

The focus groups comprised a total of 163 participants, with an average age of 41 years. These individuals represented the stakeholders and decision-makers involved in addressing the dengue fever epidemic in Hormozgan province, situated in the south of Iran. Table 2 provides an overview of the demographic characteristics of the participants.

The qualitative content analysis of the focus group discussions yielded seven main categories, which encompass determinants such as environmental, health and treatment, intersectoral cooperation, administrative-legal-regulatory, financial and budgetary, and educational/social. A detailed description of each category can be found in Table 3.

**Table 3:** Categories resulting from focus group discussions.

Categories	Sub-categories
Environmental determinants	1- Environmental-sanitary improvement of buildings 2- Application of biological control methods for vectors 3- Identification and grading of critical points 4- Environmental improvement of ports and beaches 5- Identification and removal of worn-out boats and tires 6- Collection of environmental waste 7- Repairs of drinking water networks 8- Elimination of reedbeds 9- Dredging of canals within the city
Therapeutic and health care determinants	1- Establish and implement a syndromic surveillance system for dengue fever control 2- Form and dispatch specialist treatment teams to infected areas 3- Carry out research and seroepidemiology projects 4- Create discounted treatment tariffs 5- Increase the capacity of hospitals and medical staff 6- Hold new treatment webinars
Interdisciplinary Cooperation determinants	1- Cooperation between relevant departments 2- Development and implementation of new interdisciplinary guidelines 3- Conclusion of interdisciplinary memoranda of understanding
Administrative, legal, and regulatory determinants	1- Establish and enforce strict laws 2- Monitor and control free trade zones
Financial and budgetary determinants	1- Cost estimation and required budget
Educational determinants	1- Informing the community through educational campaigns 2- Training of stakeholders
Social determinants	1- Attracting social participation 2- Health education and health promotion 3- Implementing the Aedes mosquito-free neighborhood action plan 4- Regular and periodic review of people’s challenges

### Environmental determinants

The first and most important determinant that was widely and repeatedly discussed by the participants was the contributing factor related to environmental features. The main environmental area was discussed by the environmental improvement committee, focusing on what can be done to control the population of *Aedes* mosquitoes. The 9 subcategories of this category are presented below. Environmental-sanitary improvement of buildings, application of biological control methods for vectors, identification and grading of critical points, environmental improvement of ports and beaches, identification and removal of worn-out boats and tires, collection of environmental waste, repairs of drinking water networks, elimination of reedbeds, dredging of canals within the city. The issues raised in this area from the perspective of the participants in the focus group discussions are as follows.

- *The presence of waste and construction debris in urban roads*,* neighborhoods*,* and outskirts has prompted municipalities to implement measures for the proper transfer of this waste to designated disposal sites. Additionally*,* it is essential to establish effective mechanisms to prevent the accumulation of construction waste and debris on roads and in residential areas* (Participant 2).
- *The engineering system organization must perform a thorough inspection to ensure that elevator pits in buildings under construction are not utilized for storage*,* the bottom of the elevator pit is sloped*,* and the wells dug for the building are properly covered* (Participant 7).
-*The University of Medical Sciences in the province*,* working alongside the local municipalities*,* ought to employ biological techniques*,* like introducing the western mosquitofish (Gambusia affinis) into urban water bodies or other biological methods*,* to combat the Aedes mosquito* (Participant 6).
- *It is essential for municipalities to identify the critical locations in the province where the Aedes mosquito is prevalent. This can be achieved through the prioritization and color-coding scheme developed by the university (red for immediate attention within 3 days*,* orange within 10 days*,* and yellow within a month)*,* in coordination with city health centers* (Participant 43).
- *Due to the presence of worn tires in commercial and passenger ports*,* the municipalities of coastal cities should take action and report the results regarding the collection and disposal of said tires to prevent mosquitoes from laying eggs in these tires* (Participant 10).
- *In light of the numerous abandoned boats found along the coastlines in certain regions of Bandar-Sirik city (including Bandar-Sirik*,* Bandar-Grog*,* and Bandar-Kohestak)*,* it is imperative for the municipalities*,* in collaboration with the local health center*,* to promptly remove these vessels. Additionally*,* the collection and proper disposal of discarded tires should be prioritized. These actions are essential to mitigate the risk of Aedes mosquito breeding and the potential for larval infestations or myiasis* (Participant 4).
- *Considering the possibility of Aedes mosquito transmission through cargo and passenger barges*,* according to a letter*,* the owners of such vessels are required to periodically spray their vessels with the coordination of the university health department* (Participant 12).
- *Repairs and improvements of the worn-out drinking water network in the context and old neighborhoods of the cities should be carried out through the water and sewerage company of the monitored province* (Participant 3).
- *It is crucial to promptly address the collection of construction debris in urban areas*,* as they serve as breeding grounds for Aedes mosquitoes* (Participant 14).
- *Given that the reed fields within the estuaries of urban areas serve as conducive environments for the spawning*,* development*,* and propagation of the Aedes mosquito*,* it is imperative for the relevant environmental and municipal authorities to eradicate the reeds and consistently maintain a schedule for the thorough cleansing of the estuaries and canals* (Participant 9).
- *Due to the lack of proper slope in the channels for collecting and directing surface water in the cities of the province*,* the municipality should be monitored in cooperation with the regional water company by conducting field surveys and taking necessary technical measures to solve the challenge* (Participant 17).

### Therapeutic and health care determinants

The participants frequently emphasized another determinant concerning community health controls, patient treatment, and care of individuals under suspicion. The 6 subcategories of this category are presented below. Establish and implement a syndromic surveillance system for dengue fever control, form and dispatch specialist treatment teams to infected areas, carry out research and seroepidemiology projects, create discounted treatment tariffs, increase the capacity of hospitals and medical staff, hold new treatment webinars. The quotes from the participants are as follows:

- *It is essential to prioritize the establishment of specific clinical teams for the development of a dengue syndrome care system* (Participant 31).
- *It is important to conduct Seroepidemiological research projects in a focused approach*,* specifically targeting groups with a higher risk of being bitten by Aedes mosquitoes* (Participant 37).
- *Dispatching a cohort of expert physicians and laboratory staff to the areas experiencing an outbreak of the disease* (Participant 34).
- *It is essential to prioritize the identification of 10–20 centers across the province for the referral and sampling of high-risk individuals* (Participant 38).
-*Sending samples of Aedes aegypti larvae and mosquitoes from the cities to the reference laboratory to investigate mosquito contamination* (Participant 33).
-*Setting up 24-hour medical centers in all the cities of the province to diagnose people suspected of dengue fever* (Participant 40).
-*Preparation and distribution of the treatment protocol for diseases transmitted by invasive Aedes by the Department of Infectious Diseases of Hormozgan University of Medical Sciences* (Participant 32).
- *It may be beneficial to explore the option of offering a discounted price for molecular tests used in diagnosing dengue fever* (Participant 43).
-*Creating a special meeting to prepare for a possible epidemic of dengue fever in the presence of the governor* (Participant 35).
- *Allocation of additional hospitalization capacity within healthcare facilities through the utilization of public and underutilized areas for the admission of patients affected by dengue fever* (Participant 41).
-*Prioritizing the care and fight against Aedes mosquitoes according to the revisions made in the entire province* (Participant 30).
-*Holding an educational webinar to justify the focal point and other forces involved in the program in subordinate cities* (Participant 39).

### Interdisciplinary cooperation determinants

Another key determinant, mentioned in nearly all topics and focus groups, was interdisciplinary cooperation among relevant departments and community stakeholders. The 3 related subcategories of this category are: cooperation between relevant departments, development and implementation of new interdisciplinary guidelines, conclusion of interdisciplinary memoranda of understanding. Participants in the focus group discussions raised the following issues in this area.

- *The municipalities of the coastal cities of the province*,* while coordinating with the fisheries departments of the relevant city*,* should put the necessary measures on their agenda regarding overturning inactive fishing boats* (Participant 50).
- *The General Administration of Roads and Urban Development of the province*,* the Organization of Construction Engineering System*,* and the General Administration of Standards should investigate such matters as the surface of the inclined elevator pit to prevent water from accumulating and pump it easily*,* and the General Administration of Standards of the province should take action on projects Determining an inactive and stopped building (with writing the banners*,* warning*,* and improvement by the municipality and then the cost of improvement should be included in the file of the building unit)* (Participant 59).
- *The Ministry of Energy*,* in cooperation with the municipalities*,* should remove the reeds and clean the streams and canals continuously and periodically* (Participant 53).
-*Conclusion of a memorandum of understanding between all departments*,* including the General Department of Environmental Protection*,* the provincial health network*,* municipalities the University of Medical Sciences*,* and other departments in implementing the dengue fever prevention plan* (Participant 62).

### Administrative, legal, and regulatory determinants

The next determinant involved the design, approval, notification, and implementation of guidelines for controlling the *Aedes* mosquito population and preventing dengue fever, engaging all relevant departments and stakeholders. It is crucial to address departments and individuals who disregard these guidelines. Two subcategories of this category are establishing and enforcing strict laws and monitor and control free trade zones. The focus group discussions raised the following issues in this regard.

-*To expedite action and manage the necessary time for addressing Aedes*,* the University of Medical Sciences should send original correspondence to the Bandar Abbas municipality via courier directly to the deputy of municipal services. Similarly*,* correspondence from other municipalities should be delivered to the mayor by the city health centers* (Participant 76).
-*All correspondence should be sent simultaneously to the General Department of Urban Affairs and Councils for follow-up* (Participant 70).
-*Obtaining a court order to enter seized buildings to improve units is done by the Bandar Abbas Municipality* (Participant 81).
-*Report on actions taken for entry into foreclosed buildings to enhance the units*,* submitted to the General Department of Urban Affairs*,* Bandar Abbas Municipality* (Participant 83).
- *Notification of protective instructions to control airline lines and commercial-free zones to control the mosquito population* (Participant 71).
*- Monitoring the prohibition of using elevator shafts as water storage tanks at construction sites*,* per Article 33 of the Engineering System Law*,* under the Ministry of Roads and Urban Development’s supervisory duties* (Participant 74).
- *Considering that in some residential complexes and… in Bandar Abbas city*,* the water from the cooling devices of the buildings is transported to the side of the roads and left after collection*,* it is necessary to oblige the owners of new buildings to transport water Referred to as the sewage well of the complex or the green spaces on the edge of such buildings*,* neighborhood by neighborhood information should be given and in case of non-cooperation*,* a warning should be given* (Participant 79).
-*Municipalities should take action to drain stagnant water from ponds and fountains in parks and public places and wash such ponds every week* (Participant 77).
-*Bandar Abbas municipality should act to address the remaining inactive fishing boats; of the 1*,*244 identified*,* 1*,*091 have been handled so far* (Participant 75).

### Financial and budgetary determinants

The next key determinant was financial and budgetary factors. Implementing actions, particularly at the macro level across the province and involving various departments, necessitates budgetary support. Thus, this determinant is fundamental to executing all proposed activities. The only subcategory of this category is cost estimation and required budgeting. The issues raised in this area, as expressed by participants in the focus group discussions, are as follows.

- *The municipalities of the coastal cities of the province should prepare and send to the University of Medical Sciences and Health Services of the province regarding the review and declaration of credit required for expenses* (Participant 101).
- *Cost estimation should be done in case of a dengue fever epidemic* (Participant 112).

### Educational determinants

The next determinant focused on educating community members about the dengue fever disease vector, its causative agent, and effective strategies to control the *Aedes* mosquito population. Two subcategories of this category are informing the community through educational campaigns and training of stakeholders. The participants in the focus group discussions raised the following issues.

-*The University of Medical Sciences and Health Services of the province should send training files on fighting Aedes mosquitoes to the Building Engineering System Organization and the mayors of coastal cities*,* for uploading on the relevant sites and installing on billboards*,* for information* (Participant 121).
-*Organizing monthly and continuous educational meetings in city committees with the presence of all stakeholders by the University of Medical Sciences of the province to teach effective and up-to-date materials and solutions for other countries or provinces* (Participant 125).
-*Sending instructions to relevant departments and organizations and educating the public through the province’s television and radio media* (Participant 132).

### Social determinants

The final determinant involved social factors crucial for controlling the *Aedes* mosquito population and preventing dengue fever. The key factor in this category was community participation, as residents play the most significant role in managing the mosquito population. four subcategories of this category are attracting social participation, health education and health promotion, implementing the *Aedes* mosquito-free neighborhood action plan, regular and periodic review of people’s challenges. From the participant’s perspective, the issues raised are as follows:

-*Aedes-free city initiative involving education*,* health promotion*,* environmental and occupational health*,* and community participation* (Participant 161).
-*Implementation of the Aedes-free neighborhood action plan based on the risk communication and social participation action plan with the communication approach for development* (Participant 152).
-*Weekly and monthly monitoring plan with the cooperation of citizens to check the implementation process of the program and challenges* (Participant 155).
- *Conducting training sessions for peer groups on preventing Aedes mosquito bites and the diseases they transmit* (Participant 159).
-*Creating a channel or group in virtual space by personnel and employees to* transmit educational messages to citizens (Participant 150).
- It is recommended to improve the workplace according to the checklist sent by the health deputy to receive an Aedes-free workplace certificate with the cooperation of business owners and small businesses (Participant 157).

## Discussion

This study aimed to develop a comprehensive framework for dengue prevention in Iran’s newly endemic regions by identifying key determinants through multi-stakeholder engagement. Rather than focusing solely on vector control, we sought to understand how ecological, administrative, and social factors interact in Iran’s unique context, where climate change and international travel create emerging vulnerabilities.

### Environmental determinants

The study revealed that environmental factors were the most frequently emphasized determinant, with participants highlighting issues such as waste accumulation in urban areas, stagnant water in construction sites, and neglected coastal zones with abandoned boats and tires. These findings align with global studies demonstrating the direct link between poor environmental management and Aedes proliferation^35^. In Hormozgan province, the combination of a tropical climate and high maritime activity creates ideal breeding conditions, necessitating urgent interventions such as weekly municipal cleanups and enforced slope standards for drainage systems. To operationalize these findings, we recommend implementing a ‘Color-Coded Zone Prioritization System’, inspired by successful models in Australia^18^, where high-risk areas (e.g., ports) are sanitized within 72 h (red zones), while lower-risk areas follow monthly^36^. Additionally, biological controls like Gambusia affinis fish could be introduced in urban water bodies, as successfully piloted in Thailand^37^. A critical policy gap is the lack of climate adaptation strategies; future research should model how rising temperatures may expand Aedes habitats to northern Iran.

### Therapeutic and healthcare determinants

Participants identified gaps in diagnostic capacity, treatment affordability, and hospital preparedness, particularly in rural areas. This mirrors challenges observed in Indonesia, where decentralized testing facilities improved early detection^38^. Our study suggests that 24/7 fever clinics with subsidized molecular testing could reduce delays in diagnosis, especially for travelers from endemic regions like the UAE. Furthermore, the shortage of trained healthcare workers underscores the need for mandatory dengue management modules in medical curricula and monthly webinars for frontline staff^39^.To address cost barriers, we propose a tiered pricing model for PCR tests, where low-income patients pay 50% of the tariff, funded by provincial health budgets. A notable finding was the demand for Sero epidemiological studies in high-risk occupational groups (e.g., fishermen, port workers); such data could refine targeted surveillance^40^.

### Interdisciplinary cooperation

The lack of coordination between sectors (e.g., municipalities, fisheries, and health departments) emerged as a systemic barrier, echoing similar challenges in post-Haiyan Philippines^41^. Our results advocate for a legally binding memorandum of understanding (MoU) between all stakeholders, enforced by the provincial governor’s office, to mandate weekly cross-sector meetings and joint audits. For instance, the Fisheries Department could be required to report abandoned boats to municipalities within 48 h, while the University of Medical Sciences provides technical guidance on larval control. A replicable model is Thailand’s “Vector Control Command Center,” which reduced dengue cases by 30% through real-time data sharing^42^. Future studies should evaluate the cost-effectiveness of such models in Iran’s bureaucratic context.

### Administrative, legal, and regulatory determinants

Our findings highlight systemic weaknesses in enforcing existing public health regulations, particularly regarding construction site inspections and port surveillance. Participants unanimously advocated for stricter penalties for violations related to stagnant water accumulation - a measure proven effective in Singapore’s dengue control program where similar legal deterrence reduced mosquito breeding sites^43^. The study particularly emphasizes the vulnerability of free trade zones as potential entry points for infected vectors, necessitating immediate implementation of mandatory insecticide spraying protocols for all incoming cargo vessels. To institutionalize accountability, we recommend publishing quarterly compliance reports for each sector on the provincial health department’s website. A pilot program in Bandar Abbas could test these measures before national rollout.

### Financial and budgetary determinants

Budget constraints limited larval control programs and community outreach. Drawing from Indonesia’s experience, we propose earmarked dengue funds comprising municipal sanitation budgets (for prevention)^44^. Cost-sharing agreements with private sector partners (e.g., shipping companies) could further alleviate financial burdens. For example, the Ports Authority might Fund a portion of mosquito surveillance in exchange for reduced outbreak-related trade disruptions. A cost-benefit analysis of these models is needed to guide policymakers.

### Educational determinants

Low awareness, especially among elderly and rural populations, hindered prevention efforts. Successful campaigns in Malaysia suggest that culturally tailored messaging delivered via local radio, mosque announcements, and school workshops could improve knowledge^37^. We advocate for a “Train-the-Trainer” program where community health workers educate neighborhood leaders, creating a ripple effect. Additionally, integrating dengue modules into primary school science curricula could foster long-term behavioral change.

### Social determinants

Community participation was pivotal but inconsistent. The Aedes-Free Neighborhood initiative, where villages compete for certifications and small grants, boosted engagement in pilot areas. This mirrors Brazil’s participatory model^36^. To sustain participation, we recommend monthly town halls to address community concerns and recognize top-performing neighborhoods in provincial media. Gender-specific strategies (e.g., women-led cleanup teams) could also enhance inclusivity, as seen in Indonesia^45^.

## Limitations and strengths of the study

One of the limitations of this study was the absence of a comprehensive perspective among most meeting attendees regarding the adoption of the focus group approach. Moreover, encountering challenges in engaging key individuals with availability and willingness to contribute was a notable hindrance. To address this limitation and encourage participation, emphasis was placed on elucidating the issue’s significance to community health, coupled with the provision of incentives. Another constraint is associated with the intrinsic nature of qualitative inquiry, restricting the generalizability of findings due to variations influenced by health, socio-economic, environmental, cultural, and geographical contexts. To overcome this limitation, mixed-method and interventional studies can be suggested below in Hormozgan and other provinces with similar experienced problems.

## Future studies

The outcomes derived should be leveraged to enhance similar scenarios, curtail disease transmission, and elevate the standards of healthcare delivery, treatment, surveillance, and strategic planning within dengue fever management initiatives. Given the fact that quantitative methods are usually unable to identify and explain in depth these complex dimensions and interactions of dengue fever control, while the qualitative method allowed us to gain a deeper and more comprehensive understanding of the experiences, perspectives and solutions recommendations include devising model-based interventions based on the research outcomes to mitigate challenges in subsequent studies. It is essential that future studies include a diverse set of senior managers from key sectors and participating stakeholders who will help formulate and implement critical decisions to advance public health. Such studies can be distinguished by gaining first-hand insight and expert experience.

## Conclusion

Given the escalating prevalence of re-emerging diseases globally, identifying a multitude of determinants encompassing health, environmental, social, economic, regulatory, as well as cultural, and political aspects can significantly facilitate disease management in a specific area promptly, thereby averting a potential global pandemic. The utilization of the focus group methodology in Iran during 2024 exemplified the efficacy of diverse elements, particularly the enhancement of environmental conditions and community engagement in combatting dengue fever. Future research should focus on community-based interventions, including the use of behavior change models in the community, environmental and interdisciplinary interventions, and the creation of participatory campaigns. Collaboration between experts in the fields of epidemiology, health education and health promotion, environmental health, and vector control for the prevention and control of dengue fever in the country and similar fields around the world should be considered by researchers and stakeholders.

## Data Availability

Data can be inquired from the corresponding author.

## References

[1] Hasan, S., Jamdar, S. F., Alalowi, M. & Al Ageel Al Beaiji, S. M. Dengue virus: A global human threat: review of literature. *J. Int. Soc. Prev. Community Dent.* **6** (1), 1–6 (2016). 27011925 10.4103/2231-0762.175416 PMC4784057

[2] Bhatt, S. et al. The global distribution and burden of dengue. *Nature* **496** (7446), 504–507 (2013). 23563266 10.1038/nature12060 PMC3651993

[3] Staples, K., Neville, P. J., Richardson, S. & Oosthuizen, J. Development of a regional climate change model for Aedes vigilax and Aedes Camptorhynchus (Diptera: Culicidae) in perth, Western Australia. *Bull. Entomol. Res.* **114** (1), 8–21 (2024). 38235528 10.1017/S0007485323000561

[4] Abbasi, E. The impact of climate change on Aedes aegypti distribution and dengue fever prevalence in semi-arid regions: A case study of Tehran province, Iran. *Environ. Res.* **275**, 121441 (2025). 40118318 10.1016/j.envres.2025.121441

[5] Narayanasami, E., Umakanth, M. & Suganthan, N. Dengue hemorrhagic fever complicated with hemophagocytic lymphohistiocytosis in an adult with diabetic ketoacidosis. *Cureus* **12** (8), e10172 (2020). 33029452 10.7759/cureus.10172 PMC7529484

[6] Kirawittaya, T. et al. Evaluation of cardiac involvement in children with dengue by serial echocardiographic studies. *PLoS Negl. Trop. Dis.* **9** (7), e0003943 (2015). 26226658 10.1371/journal.pntd.0003943 PMC4520477

[7] Peng, J. et al. Biased virus transmission following sequential coinfection of Aedes aegypti with dengue and Zika viruses. *PLoS Negl. Trop. Dis.* **18** (4), e0012053 (2024). 38557981 10.1371/journal.pntd.0012053 PMC10984552

[8] Weaver, S. C., Charlier, C., Vasilakis, N. & Lecuit, M. Zika, chikungunya, and other emerging Vector-Borne viral diseases. *Annu. Rev. Med.* **69**, 395–408 (2018). 28846489 10.1146/annurev-med-050715-105122 PMC6343128

[9] Shepard, D. S., Undurraga, E. A., Halasa, Y. A. & Stanaway, J. D. The global economic burden of dengue: a systematic analysis. *Lancet Infect. Dis.* **16** (8), 935–941 (2016). 27091092 10.1016/S1473-3099(16)00146-8

[10] Roiz, D. et al. The rising global economic costs of invasive Aedes mosquitoes and Aedes-borne diseases. *Sci. Total Environ.* **933**, 173054 (2024). 38729373 10.1016/j.scitotenv.2024.173054

[11] Dorzaban, H. et al. Mosquito surveillance and the first record of morphological and molecular-based identification of invasive species Aedes (Stegomyia) aegypti (Diptera: Culicidae), southern Iran. *Exp. Parasitol.* **236-237**, 108235 (2022). 35247382 10.1016/j.exppara.2022.108235

[12] Hasani, S. J. et al. Recent advances in the control of dengue fever using herbal and synthetic drugs. *Heliyon* **11** (3), e41939 (2025). 40196797 10.1016/j.heliyon.2025.e41939 PMC11947709

[13] Amidi, R. & Fatemi, S. M. R. Investigation and evaluation of risk of pathogen transfer by ballast water in Shahid Rajaee port, hormozgan province, Iran. *Environ. Monit. Assess.* **196** (12), 1174 (2024). 39503815 10.1007/s10661-024-13329-z

[14] Rezza, G. Climate change and the spread of Aedes mosquito-borne viruses in Europe. *Pathog Glob Health*. **118** (4), 358–359 (2024). 38421348 10.1080/20477724.2024.2323842 PMC11234909

[15] Heydarifard, Z., Heydarifard, F., Mousavi, F. S. & Zandi, M. Dengue fever: a decade of burden in Iran. *Front. Public. Health*. **12**, 1484594 (2024). 39507666 10.3389/fpubh.2024.1484594 PMC11537875

[16] Alshammari, S. A. et al. Overview of dengue and Zika virus similarity, what can we learn from the Saudi experience with dengue fever? *Int. J. Health Sci. (Qassim)*. **12** (1), 77–82 (2018). 29623022 PMC5870307

[17] Nejati, J., Okati-Aliabad, H., Mohammadi, M., Akbari, M. & Moghaddam, A. A. Knowledge, attitudes, and practices of healthcare professionals regarding dengue fever in high-risk regions of southeastern Iran. *BMC Med. Educ.* **24** (1), 915 (2024). 39180056 10.1186/s12909-024-05923-z PMC11344303

[18] Ryan, S. J., Carlson, C. J., Mordecai, E. A. & Johnson, L. R. Global expansion and redistribution of Aedes-borne virus transmission risk with climate change. *PLoS Negl. Trop. Dis.* **13** (3), e0007213 (2019). 30921321 10.1371/journal.pntd.0007213 PMC6438455

[19] Patel, J. P., Saiyed, F. & Hardaswani, D. Dengue fever accompanied by neurological manifestations: challenges and treatment. *Cureus* **16** (5), e60961 (2024). 38910682 10.7759/cureus.60961 PMC11193856

[20] Srisawat, N. et al. Proceedings of the 6th Asia dengue summit, June 2023. *PLoS Negl. Trop. Dis.* **18** (3), e0012060 (2024). 38551892 10.1371/journal.pntd.0012060 PMC10980189

[21] Soo, W. F., Gunasekaran, K., Ng, D. X., Kwek, K. & Tan, N. C. Literacy and attitude of Asian youths on dengue and its prevention in an endemic developed community. *Front. Public. Health*. **12**, 1361717 (2024). 38528862 10.3389/fpubh.2024.1361717 PMC10962923

[22] Lee, F., Simon, K. S. & Perry, G. L. W. Increasing agricultural land use is associated with the spread of an invasive fish (Gambusia affinis). *Sci. Total Environ.* **586**, 1113–1123 (2017). 28214124 10.1016/j.scitotenv.2017.02.101

[23] Kassiri, H. et al. Insecticide resistance in urban pests with emphasis on urban pests Re-sistance in iran: A review. *Entomol. Appl. Sci. Lett.* **7** (3-2020), 32–54 (2020).

[24] Dambach, P., Louis, V. R., Standley, C. J. & Montenegro-Quiñonez, C. A. Beyond top-down: community co-creation approaches for sustainable dengue vector control. *Glob Health Action*. **17** (1), 2426348 (2024). 39514564 10.1080/16549716.2024.2426348 PMC11552243

[25] Huang, X. et al. Dynamic Spatiotemporal trends of imported dengue fever in Australia. *Sci. Rep.* **6**, 30360 (2016). 27460696 10.1038/srep30360 PMC4961948

[26] Nikookar, S. H., Fazeli Dinan, M., Zaim, M. & Enayati, A. Prevention and control policies of dengue vectors (Aedes aegypti and Aedes albopictus) in Iran. *Iran. J. Health Sci.* **11** (3), 143–156 (2023).

[27] Seyed-Khorami, S. M. et al. A comprehensive seroepidemiology of dengue and Chikungunya arboviruses in iran, 2020–2023. *Virol. J.* **21** (1), 305 (2024). 39593084 10.1186/s12985-024-02574-w PMC11590371

[28] Adhikari, B. et al. A realist review of community engagement with health research. *Wellcome Open. Res.* **4**, 87 (2019). 31289754 10.12688/wellcomeopenres.15298.1 PMC6611131

[29] Srisawat, N., Thisyakorn, U., Ismail, Z., Rafiq, K. & Gubler, D. J. World dengue day: A call for action. *PLoS Negl. Trop. Dis.* **16** (8), e0010586 (2022). 35925876 10.1371/journal.pntd.0010586 PMC9352018

[30] Krueger, R. A. *Focus Groups: A Practical Guide for Applied Research* (Sage, 2014).

[31] Breen, R. L. A practical guide to focus-group research. *J. Geogr. High. Educ.* **30** (3), 463–475 (2006).

[32] Morgan, D. L., Krueger, R. A. & King, J. A. The focus group guidebook: Sage; (1998).

[33] Abdallah, A. A. et al. A developed MEDICAL + and MEDICAL PLUS + for Tele patient care web applications. *Clin. eHealth*. **6**, 96–113 (2023).

[34] Byrne, D. A worked example of Braun and clarke’s approach to reflexive thematic analysis. *Qual. Quant.* **56** (3), 1391–1412 (2022).

[35] Buhler, C., Winkler, V., Runge-Ranzinger, S., Boyce, R. & Horstick, O. Environmental methods for dengue vector control–A systematic review and meta-analysis. *PLoS Negl. Trop. Dis.* **13** (7), e0007420 (2019). 31295250 10.1371/journal.pntd.0007420 PMC6650086

[36] Ryan, P. A. et al. Establishment of wMel Wolbachia in Aedes aegypti mosquitoes and reduction of local dengue transmission in Cairns and surrounding locations in Northern queensland, Australia. *Gates Open. Res.* **3**, 1547 (2019). 31667465 10.12688/gatesopenres.13061.1 PMC6801363

[37] Champakaew, D. et al. Repellent effect of local Indigenous knowledge-based repellent in Nakhon Si thammarat, thailand, against Aedes aegypti mosquito. *Heliyon* ;**9**(11). (2023). 10.1016/j.heliyon.2023.e21589 PMC10651505 38027675

[38] Sulistyawati, S. et al. Untapped potential: A qualitative study of a hospital-based dengue surveillance system. *Am. J. Trop. Med. Hyg.* **103** (1), 120 (2020). 32394883 10.4269/ajtmh.19-0719 PMC7356460

[39] Organization, W. H., Research SPf, D., WHODoCoNT, T. T. D., Epidemic, W. H. O. & Alert, P. *Dengue: Guidelines for Diagnosis, Treatment* (World Health Organization, 2009).

[40] Duong, V. et al. Asymptomatic humans transmit dengue virus to mosquitoes. Proceedings of the National Academy of Sciences. ;112(47):14688-93. (2015). 10.1073/pnas.1508114112 PMC4664300 26553981

[41] Aumentado, C. et al. The prevention and control of dengue after typhoon haiyan. Western Pacific surveillance and response journal. *WPSAR* **6** (Suppl 1), 60 (2015). 26767138 10.5365/WPSAR.2015.6.3.HYN_018 PMC4710066

[42] Brusich, M. et al. Targeting educational campaigns for prevention of malaria and dengue fever: an assessment in Thailand. *Parasites Vectors*. **8**, 1–14 (2015). 25612545 10.1186/s13071-015-0653-4 PMC4311424

[43] Ho, S. H. et al. Singapore’s 5 decades of dengue prevention and control—Implications for global dengue control. *PLoS Negl. Trop. Dis.* **17** (6), e0011400 (2023). 37347767 10.1371/journal.pntd.0011400 PMC10286981

[44] Sulistyawati, S., Surahma, S. A. M. & Sukesi, T. W. Understanding community involvement on dengue prevention in sleman, indonesia: A free listing approach. *J. UOEH*. **42** (3), 231–236 (2020). 32879187 10.7888/juoeh.42.231

[45] Inriani, S. R., Juanita, J. & Andayani, L. S. Community participation factors in implementing dengue fever symptoms prevention program. *Randwick Int. Social Sci. J.* **4** (2), 385–394 (2023).

